# Are educational aspirations associated with susceptibility to smoking, e-cigarette use, and smokeless tobacco use in adolescence?

**DOI:** 10.1093/eurpub/ckae107

**Published:** 2024-08-07

**Authors:** Hanna Ollila, Hanna Konttinen, Otto Ruokolainen, Sakari Karvonen

**Affiliations:** Department of Public Health and Welfare, Finnish Institute for Health and Welfare, Helsinki, Finland; Social Psychology, Faculty of Social Sciences, University of Helsinki, Helsinki, Finland; Social Psychology, Faculty of Social Sciences, University of Helsinki, Helsinki, Finland; Department of Public Health and Welfare, Finnish Institute for Health and Welfare, Helsinki, Finland; Department of Public Health and Welfare, Finnish Institute for Health and Welfare, Helsinki, Finland

## Abstract

The early socio-economic differences in smoking build on the interplay between individual-, family-, peer-, and school-related factors. The present study aimed to add knowledge to this by examining susceptibility to smoking (S-SM), electronic cigarette (e-cigarette) use (S-EC), and smokeless tobacco (snus) use (S-SN) by educational aspirations in a country with advanced tobacco control policies. National cross-sectional School Health Promotion study survey was conducted among 8.–9. grade students (av. 15-year-olds) in 2017 with no prior smoking (*n* = 47 589), e-cigarette use (*n* = 49 382), or snus use (*n* = 53 335). Gender-stratified, age-adjusted multilevel logistic regression analyses with S-SM, S-EC, and S-SN were considered as outcomes and student- and school-level (aggregated) factors were considered as independent variables. The highest prevalence was observed for S-EC (girls 29%, boys 35%), followed by S-SM (16%, 15%) and S-SN (10%, 16%). Compared to those planning for general upper secondary education, S-SM was the highest for those without educational aspirations (OR = 1.20, 95% CI = 1.04–1.40), S-EC for those planning for vocational education [1.15 (1.05–1.25)], and S-SN for those planning for extra year/discontinuation [1.65 (1.04–2.60)] among girls. Among boys, both S-SM [1.37 (1.23–1.52)] and S-EC [1.19 (1.09–1.29)] were the highest among those planning for vocational education, with no clear associations with S-SN. Current other tobacco/e-cigarette use [OR range 1.27–8.87], positive attitude towards product use in one’s age group [3.55–6.63], and liking school [0.58–0.68] consistently associated with susceptibility. Students not planning for academically oriented education had higher susceptibility to different nicotine products. High S-EC warrants monitoring to strengthen policy evaluation and prevention.

## Introduction

Nicotine affects the developing brain negatively [1]. Some adolescents experience first dependence symptoms early [2], which makes prevention of nicotine use essential. Despite the general decline in smoking, it remains more prevalent among youth with lower socio-economic position (SEP). This has been observed with family-level SEP indicators, including parental education [3, 4] and family affluence [5]as well as individual-level indicators of adulthood SEP such as academic performance [4, 5] and vocational education [6–8]. For smokeless tobacco (snus) use and electronic cigarette (e-cigarette) use, the associations with SEP have been mixed [7, 9, 10].

In Europe, all countries impose educational tracking by the age of 16, and the entrance to general or academic tracks requires students to pass or perform well [11]. This directs lower-performing students to vocational education, but in several European countries, vocational education is also a common selection in upper secondary education attracting students with diverse background [11]. Smoking predicts the selection of non-academically oriented education [12] and is associated with lower educational aspirations prior to track selection [13, 14] Similar conclusions have been found for snus use, though to a lesser extent [15, 16]. For e-cigarettes, this has not been studied. In adolescence, the interplay between educational pathways and health-related behaviours appears as one of the mechanisms leading to later health inequalities [17].

To identify youth at the highest risk of initiation, the concept of susceptibility to smoking (S-SM) was developed in the 1990s [18]. It builds on the assumption that lack of a firm commitment not to smoke, rather than intention to smoke, may determine youth behaviour if the opportunity to experiment presents. The concept and measure have been adapted and validated for non-combustible nicotine products such as e-cigarettes and smokeless tobacco [19]. Poor academic performance [20] and parental and peer smoking have been associated with increased S-SM [21], whereas school connectedness has had a protective effect [22]. Susceptibility to non-combustible product use has also been consistently more common among youth with peers using a similar product, but associations with academic performance have been inconclusive [23, 24]. The associations with gender have varied both for the S-SM and for the use of non-combustible products [21, 24, 25]. It is currently unknown how educational aspirations are associated with S-SM or use of non-combustible products. Evidence on this is needed to better prevent inequalities in health behaviours.

Several school-level factors have a role in both youth tobacco use and educational pathways. These include school rules and policies, physical environment, curriculum, discipline, school health services, school social and learning environment, student commitment to learning and school community as well as parents and the broader community [26]. Further, school influence partly occurs through the perceptions of smoking behaviour in the social network (descriptive norms) and perceptions of social network beliefs (injunctive norms), which both predict smoking initiation among youth [27]. Upon the selection of the educational track, students may already have normative perceptions of smoking in vocational schools, such as understanding of smoking as an integral and expected practice in these schools and professions [8]. These perceptions may increase S-SM and other nicotine product use, also among students with better academic performance. Student network characteristics and their associations with smoking vary between schools [28]. In smoking and school disengagement (e.g. truancy), students often choose to affiliate with similar peers [28, 29]. Smoking prevalence has been lower in schools where educational attainment and attendance are better than predicted based on student socio-demographic factors, indicating the importance of positive school ethos [30]. Yet, other school-level factors than school connectedness [22] have rarely been studied in connection with susceptibility.

Susceptibility to smoking or non-combustible nicotine use is also influenced by national tobacco control policies. In Europe, Finland has been among the countries with most advanced tobacco control regulations [31]. Still, there is a large difference in smoking and snus use between students in academically and non-academically oriented education [7]. For snus, Finland follows the EU-wide sales ban but cross-border traveller imports from Sweden are permitted and an illicit market exists [32]. For e-cigarettes, comprehensive regulations were introduced in 2016 including retail sale licensing and ban for distance sales, display and advertising at point-of-sale, other flavours than tobacco and use of e-cigarettes in smoke-free places [33]. To our knowledge, no studies in Europe have examined susceptibility to e-cigarette (S-EC) use and snus use (S-SN).

The present study aims to address the previous gaps by studying the following research questions among comprehensive school 8.–9. grade students in Finland:

- How common are S-SM, S-EC, and S-SN?
- How are educational aspirations associated with S-SM, S-EC, and S-SN at individual and school level, when examined in the context of parental education and smoking, liking school, attitudes towards smoking or non-combustible nicotine use in one’s age group, and current other nicotine product use?

## Methods

### Data and participants

National School Health Promotion (SHP) study data from 8.–9. grades in comprehensive schools in 2017 (April–May) were utilized. All comprehensive schools are invited to the SHP and 63% (*N* = 73 680) of all Finnish 8th and 9th graders participated in 2017 [34]. The SHP is an anonymous and voluntary classroom survey utilizing an online questionnaire. The students were informed of the aim and content of the survey and had an opportunity to decline participation, and their guardians were informed and could opt out of the child’s participation. The SHP study protocol has been approved by the Ethical Review Board of the Finnish Institute for Health and Welfare.

As the present study aimed to examine school-level factors, schools with less than 10 participants were excluded, forming the first dataset (*n* = 73 509, *n* = 688 schools). Three subsets were then formed: those who had responded to the questions on susceptibility and had never smoked (*n* = 47 589), had never used e-cigarettes (*n* = 49 382), or had never used snus (*n* = 53 335). The average age of students was 15.3 (SD 0.7). The characteristics of the study population are shown in Table 1.

**Table 1.** Characteristics of the study population.

	Subset 1: Never smoking (*n* = 47 589)	Subset 2: Never e-cigarette use (*n* = 49 382)	Subset 3: Never snus use (*n* = 53 335)
	%	%	%
Gender			
Girl	54.2	56.2	57.7
Boy	45.3	43.3	41.7
Missing	0.5	0.5	0.5
Educational aspirations			
General upper secondary education (GUSS)	66.0	64.4	63.7
Vocational education	24.2	25.8	26.2
Extra year/discontinuation	0.6	0.7	0.7
Does not know	8.6	8.5	8.8
Missing	0.6	0.6	0.6
At least one parent has high education			
No	42.4	43.4	44.0
Yes	46.8	45.7	45.1
Missing	10.8	10.8	10.9
At least one parent smokes			
No	74.4	72.9	72.3
Yes	22.6	24.4	25.0
Missing	3.0	2.8	2.8
Positive attitude towards smoking in one’s age group			
No	87.3	83.6	82.8
Yes	11.2	15.0	15.7
Missing	1.6	1.4	1.5
Positive attitude towards e-cigarette use in one’s age group			
No	76.2	75.7	72.1
Yes	22.1	22.8	26.3
Missing	1.7	1.5	1.6
Positive attitude towards snus use in one’s age group			
No	84.7	82.2	83.1
Yes	13.8	16.5	15.4
Missing	1.5	1.3	1.4
Ever use of tobacco product/e-cigarette^a^			
No	80.5	77.5	72.0
Yes	16.7	21.5	25.6
Missing	2.8	1.1	2.4
Current tobacco smoking^a^			
No		94.3	94.0
Yes		5.1	5.4
Missing		0.5	0.6
Current e-cigarette use^a^			
No	94.7		93.3
Yes	2.4		3.7
Missing	2.9		3.0
Current snus use^a^			
No	97.7	96.4	
Yes	1.5	2.9	
Missing	0.8	0.6	

Table displays the percentage distribution in the three subsets (never-smokers, never e-cigarette users, and never snus users) by respondents’ gender, educational aspirations, parental education, parental smoking, positive attitudes towards use in one’s age group, ever use of tobacco product or e-cigarette, and current smoking or e-cigarette use or snus use.

a Any use of the respective product is excluded from the subset.

### Outcome measures

The measures for the *S-SM, S-EC, and S-SN* were adapted from the two-question S-SM index used in the Global Youth Tobacco Survey, originally developed by Pierce et al. [35]. The questions were ‘If one of your best friends were to offer you [product], would you use it?’ and ‘Do you think it is likely that you will use [product] during the next 12 months?’ Students providing any other response except ‘Definitely not’ to either of these questions were encoded as susceptible.

### Student-level independent variables

*Educational aspirations* were measured with ‘Where do you primarily want to go to study after comprehensive school?’, including four categories (1) general upper secondary school (GUSS), (2) vocational education (combined from response options including vocational school, apprenticeship training, and vocational education with upper secondary school courses), (3) extra year or discontinuation (combined due to small number of responses for 10th grade and not intending to study any more), and (4) don’t know. *High level of parental education* was operationalized as at least one parent having tertiary education, and *parental smoking* as at least one parent smoking currently. *Liking school* was asked with ‘How do you like school at this moment?’ and dichotomized from responses Very much/quite a lot (vs. fairly little/not at all).

*Positive attitude towards product use in one’s age group* was operationalized for each product from ‘Yes’ response to ‘People have differing views on what is acceptable and what is not. Do you find the following acceptable for people of your age [Smoking/E-cigarette use/Snus use]?’ *Current smoking* was formed from Daily/Weekly/Less than weekly responses to ‘Which of the following alternatives best describes your current smoking habits?’, with the pre-condition of having smoked over 50 cigarettes, pipefuls, or cigars in lifetime. *Current e-cigarette use* was formed from ‘Daily/Now and then’ responses to ‘Do you smoke e-cigarettes that contain the following substances [Nicotine/Tobacco flavours/Other flavourings (e.g. fruit)/Other]?’ *Current snus use* was formed from ‘Daily/Now and then’ responses to ‘Have you ever used any of these [Snus]?’

### School-level variables

School-level variables were aggregated from the full dataset to provide for each school the proportion of students who (a) were girls, (b) had high levels of parental education, (c) planned to continue to GUSS, (d) liked school, (e) reported parental smoking, (f) had never smoked, used snus or e-cigarettes (never-users), and (g) had positive attitudes towards smoking/e-cigarette use/snus use in one’s age group. For the analyses, school proportions were recoded into 0–10 (1 = increase of 10 percentage points).

### Statistical analyses

Cross-tabulations and Pearson χ2-tests were conducted with IBM SPSS 27.0, and multilevel logistic regression analyses with MlwiN 3.05. Multilevel analysis was used to account for the hierarchical data. The associations between the student- and school-level independent variables and S-SM, S-EC, and S-SN were tested with two-level logistic regression models, with students on level one and schools on level two. Student class information is not available in the SHP. The estimation procedure in the random intercept models was second-order predictive quasi-likelihood (PQL2). Analyses were stratified by gender due to significant interactions between gender and educational aspirations for all three outcomes in the unadjusted analyses. Median odds ratio (MOR), which shows the difference between two respondents with identical student-level characteristics from two randomly chosen different schools, was calculated to express between-school variance on the odds ratio scale [36]. The analysis was not pre-registered, and the results should be considered exploratory.

## Results

### Descriptive results

Overall, the proportion of students with S-SM was 16% (16% girls, 15% boys, *P* < .001). For S-EC, the respective proportion was 32% (29% and 35%, *P* < .001). Altogether 13% had S-SN (10% and 16%, *P* < .001). Regardless of gender, S-SM, S-EC, and S-SN were least common among students planning for GUSS after the comprehensive school (Supplementary Fig. S1).

### Susceptibility to smoking

In the unadjusted analyses (Table 2), of the student-level factors, all other educational aspirations compared with planning for GUSS were associated with higher S-SM. This applied for both girls and boys. Similar association was found for parental smoking, positive attitude towards smoking in one’s age group, current e-cigarette use, and snus use. High parental education and liking school lowered S-SM. At school level, a higher proportion of students with positive attitudes towards smoking in one’s age group increased S-SM both among girls and boys, whereas a higher proportion of students planning for GUSS, liking school, and being never-users lowered it. Among boys, a higher proportion of students with high parental education also lowered S-SM.

**Table 2.** The association between student- and school-level factors and susceptibility to smoking.

	Girls				Boys			
	Unadjusted		Adjusted		Unadjusted		Adjusted	
	OR (95% CI)	*P*	OR (95% CI)	*P*	OR (95% CI)	*P*	OR (95% CI)	*P*
Student-level factors								
High parental education	0.87 (0.81–0.94)	<.001	1.00 (0.92–1.09)	.974	0.87 (0.81–0.95)	.001	1.04 (0.94–1.15)	.418
Educational aspirations (ref. GUSS)								
Vocational education	1.37 (1.26–1.48)	<.001	1.09 (0.98–1.21)	.102	1.65 (1.53–1.79)	<.001	1.37 (1.23–1.52)	<.001
Extra year/discontinuation	2.05 (1.38–3.04)	<.001	0.81 (0.45–1.46)	.486	3.18 (2.22–4.54)	<.001	1.17 (0.68–2.02)	.566
Does not know	1.51 (1.34–1.69)	<.001	1.20 (1.04–1.40)	.014	1.27 (1.11–1.44)	<.001	1.22 (1.03–1.44)	.022
Likes school	0.49 (0.46–0.53)	<.001	0.59 (0.54–0.64)	<.001	0.55 (0.51–0.60)	<.001	0.68 (0.61–0.74)	<.001
Parental smoking	1.38 (1.28–1.48)	<.001	1.19 (1.09–1.30)	<.001	1.23 (1.12–1.34)	<.001	1.00 (0.89–1.11)	.933
Positive attitude towards smoking in one's age group	5.92 (5.42–6.46)	<.001	5.42 (4.90–5.98)	<.001	4.21 (3.85–4.60)	<.001	3.55 (3.19–3.94)	<.001
Current e-cigarette use	3.85 (3.02–4.91)	<.001	2.18 (1.61–2.96)	<.001	3.52 (3.05–4.07)	<.001	1.99 (1.66–2.39)	<.001
Current snus use	4.94 (3.22–7.59)	<.001	1.78 (1.01–3.15)	.046	4.23 (3.59–4.98)	<.001	2.37 (1.91–2.94)	<.001
School-level factors								
Proportion of girls in the school	0.99 (0.94–1.04)	.656	1.06 (0.99–1.14)	.077	0.98 (0.93–1.04)	.596	0.99 (0.92–1.06)	.770
Proportion of students with high parental education	0.98 (0.96–1.01)	.150	1.03 (0.98–1.09)	.265	0.96 (0.93–0.98)	.001	0.95 (0.90–1.00)	.067
Proportion of students intending to continue to GUSS	0.97 (0.95–1.00)	.020	1.00 (0.94–1.05)	.855	0.97 (0.94–0.99)	.010	1.08 (1.02–1.15)	.007
Proportion of students liking school	0.92 (0.88–0.95)	<.001	1.03 (0.97–1.09)	.318	0.94 (0.90–0.98)	.003	1.01 (0.95–1.07)	.710
Proportion of students with parental smoking	1.04 (1.00–1.08)	.067	0.98 (0.91–1.04)	.502	1.06 (1.02–1.11)	.007	0.98 (0.92–1.06)	.671
Proportion of students with positive attitude towards smoking in one's age group	1.16 (1.11–1.20)	<.001	1.05 (0.98–1.14)	.186	1.15 (1.10–1.21)	<.001	1.04 (0.96–1.13)	.341
Proportion of never-users in school	0.88 (0.84–0.91)	<.001	0.91 (0.85–0.97)	.005	0.89 (0.85–0.93)	<.001	0.97 (0.90–1.04)	.421
Random part								
MOR			1.28				1.15	
Level 1 units (students)			21 967				17 283	
Level 2 units (schools)			680				680	

Table displays the results from unadjusted and adjusted two-level models. Unadjusted analyses are univariate two-level logistic regression models including associations for each independent variable. Adjusted analysis is a multivariate two-level logistic regression model including all the listed variables and age. School-level factors are continuous variables. In these, the percentages have been recoded from 0–100 to 0–10, where 1 marks an increase of 10 percentage points. Null model: Girls (*n* = 25 772, 683 schools) ICC = 0.020, MOR = 1.28; Boys (*n* = 21 562, 684 schools) ICC = 0.010, MOR = 1.19. Number of units is lower in following models due to variable non-response. CI, confidence interval; GUSS, general upper secondary education; MOR, median odds ratio; OR, odds ratio; *P*, *P* values.

In the model including all the student- and school-level factors (Table 2), S-SM remained higher for those without educational aspirations both among girls and boys. Among boys, those planning for vocational education also had higher S-SM. For both girls and boys, S-SM remained lower for those who liked school. Positive attitudes towards smoking in one’s age group were associated with higher S-SM, as was current e-cigarette use and snus use. Parental smoking associated with higher S-SM among girls. At school level, a higher proportion of never-users lowered S-SM among girls. Among boys, a higher proportion of students planning for GUSS increased it. Between-school differences were larger for girls (MOR 1.28) than for boys (MOR 1.15).

### Susceptibility to e-cigarette use

In the unadjusted analyses (Table 3), those planning for vocational education or extra year/discontinuation had higher S-EC compared with those planning for GUSS. Among girls, those without educational aspirations also had higher S-EC. Parental smoking, positive attitude towards e-cigarette use in one’s age group, current smoking, and snus use increased S-EC, whereas liking school lowered it. Among girls, higher parental education also lowered S-EC. At school level, both among girls and boys, a higher proportion of students liking school and being never-users lowered S-EC, whereas a higher proportion of students with positive attitudes towards e-cigarette use increased it. Among girls, a higher proportion of girls in the school, students with a high parental education and planning for GUSS lowered S-EC, but a higher proportion of students with parental smoking increased it.

**Table 3.** The association between student- and school-level factors and susceptibility to e-cigarette use.

	Girls				Boys			
	Unadjusted		Adjusted		Unadjusted		Adjusted	
	OR (95% CI)	*P*	OR (95% CI)	*P*	OR (95% CI)	*P*	OR (95% CI)	*P*
Student-level factors								
High parental education	0.81 (0.76–0.85)	<.001	1.01 (0.94–1.09)	.711	0.97 (0.91–1.03)	.309	1.05 (0.98–1.14)	.171
Educational aspirations (ref. GUSS)								
Vocational education	1.71 (1.61–1.82)	<.001	1.15 (1.05–1.25)	.002	1.56 (1.47–1.66)	<.001	1.19 (1.09–1.29)	<.001
Extra year/discontinuation	2.31 (1.70–3.14)	<.001	1.22 (0.79–1.90)	.366	2.00 (1.48–2.70)	<.001	0.85 (0.56–1.31)	.472
Does not know	1.37 (1.24–1.50)	<.001	1.13 (0.99–1.28)	.072	1.05 (0.95–1.16)	.339	1.01 (0.88–1.15)	.934
Likes school	0.43 (0.41–0.45)	<.001	0.58 (0.54–0.62)	<.001	0.50 (0.47–0.53)	<.001	0.63 (0.58–0.68)	<.001
Parental smoking	1.70 (1.61–1.80)	<.001	1.24 (1.15–1.34)	<.001	1.41 (1.32–1.50)	<.001	1.10 (1.01–1.20)	.022
Positive attitude towards product use in one’s age group	8.59 (8.05–9.17)	<.001	6.63 (6.15–7.14)	<.001	5.51 (5.15–5.88)	<.001	4.62 (4.28–4.98)	<.001
Current smoking	17.59 (15.22–20.32)	<.001	8.87 (7.43–10.59)	<.001	11.86 (9.88–14.22)	<.001	5.62 (4.46–7.08)	<.001
Current snus use	11.83 (8.76–15.97)	<.001	2.53 (1.66–3.84)	<.001	9.13 (7.86–10.62)	<.001	4.06 (3.38–4.89)	<.001
School-level factors								
Proportion of girls in the school	0.94 (0.90–0.97)	.001	0.99 (0.93–1.05)	.680	1.00 (0.96–1.04)	.963	1.05 (0.99–1.11)	.085
Proportion of students with high parental education	0.96 (0.94–0.98)	<.001	1.01 (0.97–1.06)	.600	1.00 (0.98–1.02)	.948	1.02 (0.98–1.07)	.392
Proportion of students intending to continue to GUSS	0.95 (0.94–0.97)	<.001	1.01 (0.97–1.06)	.576	0.99 (0.97–1.01)	.233	1.02 (0.97–1.07)	.463
Proportion of students liking school	0.91 (0.88–0.94)	<.001	1.04 (0.99–1.09)	.143	0.94 (0.91–0.97)	<.001	1.03 (0.98–1.07)	.308
Proportion of students with parental smoking	1.10 (1.07–1.13)	<.001	1.00 (0.94–1.05)	.856	1.02 (0.99–1.06)	.186	0.99 (0.94–1.05)	.770
Proportion of students with positive attitude towards product use in one’s age group	1.22 (1.18–1.25)	<.001	1.02 (0.96–1.07)	.576	1.15 (1.11–1.19)	<.001	1.01 (0.95–1.06)	.863
Proportion of never-users in the school	0.83 (0.81–0.86)	<.001	0.90 (0.85–0.95)	<.001	0.85 (0.82–0.87)	<.001	0.90 (0.85–0.95)	<.001
Random effects								
MOR			1.23				1.16	
Level 1 units (students)			24 083				17 537	
Level 2 units (schools)			681				684	

Table displays the results from unadjusted and adjusted two-level models. Unadjusted analyses are univariate two-level logistic regression models including associations for each independent variable. Adjusted analysis is a multivariate two-level logistic regression model including all the listed variables and age. School-level factors are continuous variables. In these, the percentages have been recoded from 0–100 to 0–10, where 1 marks an increase of 10 percentage points. Null model: Girls (*n* = 27 730, 683 schools) ICC= 0.019, MOR = 1.27; Boys (*n* = 21 401, 687 schools); ICC = 0.011, MOR = 1.20. Number of units is lower in following models due to variable non-response. CI, confidence interval; GUSS, general upper secondary education; MOR, median odds ratio; OR, odds ratio; *P*, *P* values.

In the multivariate model (Table 3), results were similar for both girls and boys. S-EC remained higher for those planning for vocational education, and lower with liking school. Further, S-EC was higher with parental smoking, positive attitudes towards e-cigarette use in one’s age group, current smoking, and current snus use. At school level, a higher proportion of never-users lowered S-EC. Between-school differences were larger for girls (MOR 1.23) than boys (MOR 1.16).

### Susceptibility to snus use

In the unadjusted analyses (Table 4), all other educational aspirations compared with planning for GUSS were associated with higher S-SN, as were also parental smoking, positive attitude towards snus use in one’s age group, current smoking, and e-cigarette use both among girls and boys. High parental education and liking school lowered S-SN. At school level, a higher proportion of students liking school and being never-users lowered S-SN, but a higher proportion of students with positive attitudes towards snus use in one’s age group increased it. Among girls, a higher proportion of girls in the school and a higher proportion of students with high parental education and planning for GUSS also lowered S-SN.

**Table 4.** The association between student- and school-level factors and susceptibility to snus use.

	Girls				Boys			
	Unadjusted		Adjusted		Unadjusted		Adjusted	
	OR (95% CI)	*P*	OR (95% CI)	*P*	OR (95% CI)	*P*	OR (95% CI)	*P*
Student-level factors								
High parental education	0.79 (0.73–0.85)	<.001	0.94 (0.86–1.04)	.222	0.92 (0.86–1.00)	.047	1.01 (0.91–1.10)	.924
Educational aspirations (ref. GUSS)								
Vocational education	1.70 (1.56–1.85)	<.001	1.15 (1.03–1.29)	.012	1.36 (1.26–1.47)	<.001	1.10 (0.99–1.22)	.067
Extra year/discontinuation	3.36 (2.41–4.68)	<.001	1.65 (1.04–2.60)	.032	2.26 (1.60–3.19)	<.001	1.12 (0.70–1.79)	.632
Does not know	1.67 (1.47–1.89)	<.001	1.26 (1.07–1.48)	.005	1.21 (1.07–1.37)	.003	1.10 (0.93–1.28)	.261
Likes school	0.44 (0.41–0.47)	<.001	0.62 (0.57–0.68)	<.001	0.53 (0.49–0.57)	<.001	0.64 (0.58–0.70)	<.001
Parental smoking	1.43 (1.32–1.55)	<.001	1.02 (0.92–1.13)	.712	1.18 (1.09–1.29)	<.001	1.03 (0.93–1.14)	.551
Positive attitude towards product use in one’s age group	6.04 (5.58–6.54)	<.001	4.47 (4.06–4.92)	<.001	4.17 (3.85–4.52)	<.001	3.63 (3.30–3.99)	<.001
Current smoking	5.99 (5.43–6.60)	<.001	2.86 (2.51–3.26)	<.001	4.94 (4.25–5.75)	<.001	2.48 (2.04–3.01)	<.001
Current e-cigarette use	3.96 (3.42–4.58)	<.001	1.27 (1.05–1.53)	.015	3.26 (2.86–3.72)	<.001	1.76 (1.49–2.08)	<.001
School-level factors								
Proportion of girls in the school	0.94 (0.89–1.00)	.040	1.00 (0.93–1.08)	.973	0.96 (0.91–1.02)	.184	0.98 (0.91–1.05)	.507
Proportion of students with high parental education	0.97 (0.95–1.00)	.022	1.02 (0.96–1.08)	.473	1.00 (0.98–1.03)	.925	0.97 (0.92–1.03)	.308
Proportion of students intending to continue to GUSS	0.96 (0.94–0.99)	.004	1.00 (0.94–1.06)	.960	1.01 (0.99–1.03)	.456	1.07 (1.01–1.13)	.027
Proportion of students liking school	0.89 (0.85–0.93)	<.001	0.99 (0.93–1.05)	.755	0.95 (0.91–0.99)	.018	1.00 (0.95–1.06)	.891
Proportion of students with parental smoking	1.04 (1.00–1.09)	.054	0.99 (0.91–1.06)	.682	0.97 (0.93–1.01)	.201	0.94 (0.88–1.01)	.080
Proportion of students with positive attitude towards product use in one’s age group	1.21 (1.16–1.27)	<.001	1.03 (0.95–1.11)	.525	1.16 (1.11–1.22)	<.001	1.10 (1.02–1.19)	.010
Proportion of never-users in the school	0.86 (0.83–0.90)	<.001	0.97 (0.90–1.04)	.347	0.93 (0.89–0.96)	<.001	1.02 (0.95–1.09)	.664
Random effects								
MOR			1.27				1.16	
Level 1 units (students)			26 129				17 973	
Level 2 units (schools)			681				682	

Table displays the results from unadjusted and adjusted two-level models. Unadjusted analyses are univariate two-level logistic regression models including associations for each independent variable. Adjusted analysis is a multivariate two-level logistic regression model including all the listed variables and age. School-level factors are continuous variables. In these, the percentages have been recoded from 0–100 to 0–10, where 1 marks an increase of 10 percentage points. Null model: Girls (*n* = 30 788, 683 schools) ICC = 0.015, MOR = 1.24; Boys (*n* = 22 266, 685 schools) ICC = 0.010, MOR = 1.19. Number of units is lower in following models due to variable non-response. CI, confidence interval; GUSS, general upper secondary education; MOR, median odds ratio; OR, odds ratio; *P*, *P* values.

In the multivariate model (Table 4), all other educational aspirations compared with planning for GUSS remained associated with higher S-SN among girls, but no clear associations were found among boys. S-SN remained lower for those who liked school. S-SN was higher with positive attitudes towards snus use in one’s age group, current smoking, and current e-cigarette use. At school level, no clear associations were found among girls. Among boys, a higher proportion of students with positive attitude towards snus use in one’s age group increased S-SN, as did now also a higher proportion of students planning for GUSS. Between-school differences were larger for girls (MOR 1.27) than for boys (MOR 1.16).

## Discussion

This study explored the prevalence of S-SM, S-EC, and S-SN among adolescents in a country with advanced tobacco control regulations. We investigated the associations between educational aspirations and susceptibility, considering parental education and smoking, liking school, attitudes towards respective product use in one’s age group, and current other tobacco/e-cigarette use on individual and school level. We found that the susceptibility was highest for e-cigarette use, followed by smoking and snus use. S-SM was more common among girls, whereas S-EC and S-SN were more common among boys.

For both girls and boys, all types of susceptibility were least common among youth planning to continue to academically oriented education after the comprehensive school. Planning for vocational education was most consistently associated with higher susceptibility, whereas planning for extra year or discontinuation, or being without educational aspirations, had more diverse associations. We are not aware of any prior studies of these associations. While high parental education was mostly related to lower susceptibility when analysed independently, no clear associations were found in the multivariate models, which is similar to an earlier study on e-cigarette susceptibility [23]. Liking school was consistently associated with lower susceptibility, which supports findings from a previous study on school connectedness [22]. Taken together, our findings bring new evidence considering smoking and non-combustible nicotine product use that educational aspirations and school engagement have an important role in the initiation process in adolescence [17].

Further, educational aspirations and school engagement are tied to student composition and school-based networks and norms, which have had different associations with smoking between schools [28]. Their associations with susceptibility are unknown. In our study, there were between-school differences, which appeared larger for girls than boys. In the univariate analyses, most school-level factors either increased or lowered susceptibility. This was especially the case among girls in S-EC and S-SN, and among boys in S-SM. In the multivariate models, most of the school-level associations were weakened. Among girls, only a higher proportion of never-users in the school remained protective for S-SM and S-EC. Among boys, the same was observed for S-EC, whereas a higher proportion of students with positive attitudes towards snus use in one’s age group increased S-SN. Interestingly, a higher proportion of students planning for general upper secondary education had lowered S-EC and S-SN in the univariate analyses but increased S-SM and S-SN in the multivariate models. This may reflect, for instance, more complex interactions between individual- and school-level factors influencing susceptibility among boys. In general, students with positive attitude towards product use in one’s age group and current use of some other tobacco or nicotine product had consistently higher susceptibility regardless of product type. These findings can reflect general openness to nicotine use, indicated by a previous study showing considerable overlap between susceptibility types [24].

The novel results for school-level factors indicate that prevention of nicotine use may be supported by facilitating nicotine-free schools through policies and programmes. This may address especially the peer selection processes, which have been observed both in school disengagement and smoking initiation [28, 29]. Further, it may influence the students’ descriptive and injunctive norms, which both predict smoking initiation among youth [27]. The most consistent predictors have been descriptive norms of parents’ and close friends’ smoking, indicating that addressing close network members might be the most effective in interventions [27]. In our study, parental smoking reported by adolescents increased all types of susceptibility when analysed independently. In the multivariate models, it did not have clear associations with S-SN and S-EC among girls. To inform schools of intervention needs, the susceptibility measure could be utilized in school surveys and relevant student services as a screening tool. Simultaneously, it would support the evaluation of existing policies and programmes and monitoring of potential counter-effects. For example, an earlier study observed that school-based programmes and policies had resulted in higher susceptibility risk at some intervention schools [37]. Effective and non-counter-productive interventions are especially important for products where susceptibility is at a high level, like e-cigarettes. The appeal of e-cigarettes to youth is explained by a mixture of influence arising from product innovations facilitating new and playful user practices, marketing embedded to contemporary digital and global youth culture, normative perceptions, and social interactions [38]. Interventions to reduce susceptibility therefore need to address product regulation and marketing as well as social influence, norms, and perceptions.

Despite the long-term decrease in smoking among youth in Finland [7], S-SM was fairly common but lower than reported from central and eastern European countries and from the USA [21, 24, 25]. Yet, while not fully comparable, the current figures are lower especially for girls than in an earlier Global Youth Tobacco Survey in Finland (girls 24%, boys 16%) [39]. This may reflect the de-normalization of smoking and changed social climate, which have followed sustained tobacco control efforts [40]. To our knowledge, no prior studies have assessed the S-EC or S-SN in Europe. In comparison to 15- to 17-year-olds in the USA, S-EC in Finland appears to be at a similar level and S-SN at a clearly higher level [24]. For e-cigarettes, this might reflect the situation where the implementation of new regulations was only in its initial phase. Future research should assess the susceptibility to e-cigarettes in Finland after the regulations were fully implemented. The higher S-SN may be explained by easy access to snus from the illicit market and potential increased visibility of snus use among adolescents, especially among boys [7, 32]. As the objective of the Finnish Tobacco Act is to end the use of tobacco and non-medical nicotine products (<5% daily use by 2030), monitoring susceptibility can support the planning and evaluation measures that contribute to achieving the goal.

In conclusion, the prevalence of susceptibility among adolescents differs by product type. Liking school and academically oriented educational aspirations appear as consistent protective factors, while the use of other tobacco or nicotine products and positive attitudes towards product use in one’s age group appear as risk factors. Differences between schools exist, but having more students with no nicotine use showed protective associations, while having more students with positive attitudes towards product use in one’s age group increased the susceptibility. Our findings add to the understanding of the processes of the unequal uptake of smoking and non-combustible nicotine product use in adolescence, yet emphasize the importance of addressing social influence and attitudes in the prevention of tobacco and nicotine use also at school level. The results of the present study suggest that both interventions tailored by educational aspirations and universal school policies are needed to reach youth at high-risk of initiation.

## Strengths and limitations

The present study utilized a nationwide school survey which covers the majority of the respective Finnish student population and a validated measure for susceptibility, adapted to cover also e-cigarettes and snus. The limitations include self-reporting, lack of class-level data and a measure on school performance, and potential bias in parental education and smoking when reported by students. Further, no causal conclusions can be made from this cross-sectional data.

## Supplementary Material

ckae107_Supplementary_Data

## Data Availability

The data underlying this article cannot be shared publicly due to the privacy of individuals that participated in the study. The Finnish Social and Health Data Permit Authority Findata grants permits to the secondary use of the SHP data. Key points Highest susceptibility was observed for e-cigarette use. Susceptibility to smoking was more common among girls, whereas susceptibility to e-cigarette use and snus use were more common among boys. Educational aspirations towards academically oriented track and liking school appear as consistent protective factors. Regardless of product, higher susceptibility appears to be closely connected to use of other tobacco or nicotine products and positive attitudes towards their use in one’s own age group. Differences between schools exist but having more students with no nicotine use showed protective associations, while having more students with positive attitudes towards use in one’s age group increased susceptibility. Utilizing the susceptibility measure in monitoring may support the planning and evaluation of preventive measures in schools and at the national level.
