# Supplementary material for: Are educational aspirations associated with susceptibility to smoking, e-cigarette use, and smokeless tobacco use in adolescence?
Source: Eur J Public Health. 2024 Aug 7;34(5):962–9. doi: 10.1093/eurpub/ckae107 (PMC11430905; doi:10.1093/eurpub/ckae107)

Supplementary figure 1. Proportion (with 95% confidence intervals) of 8. and 9. Grade students with susceptibility to smoking, susceptibility to e-cigarette use, and susceptibility to snus use by gender and educational aspirations.

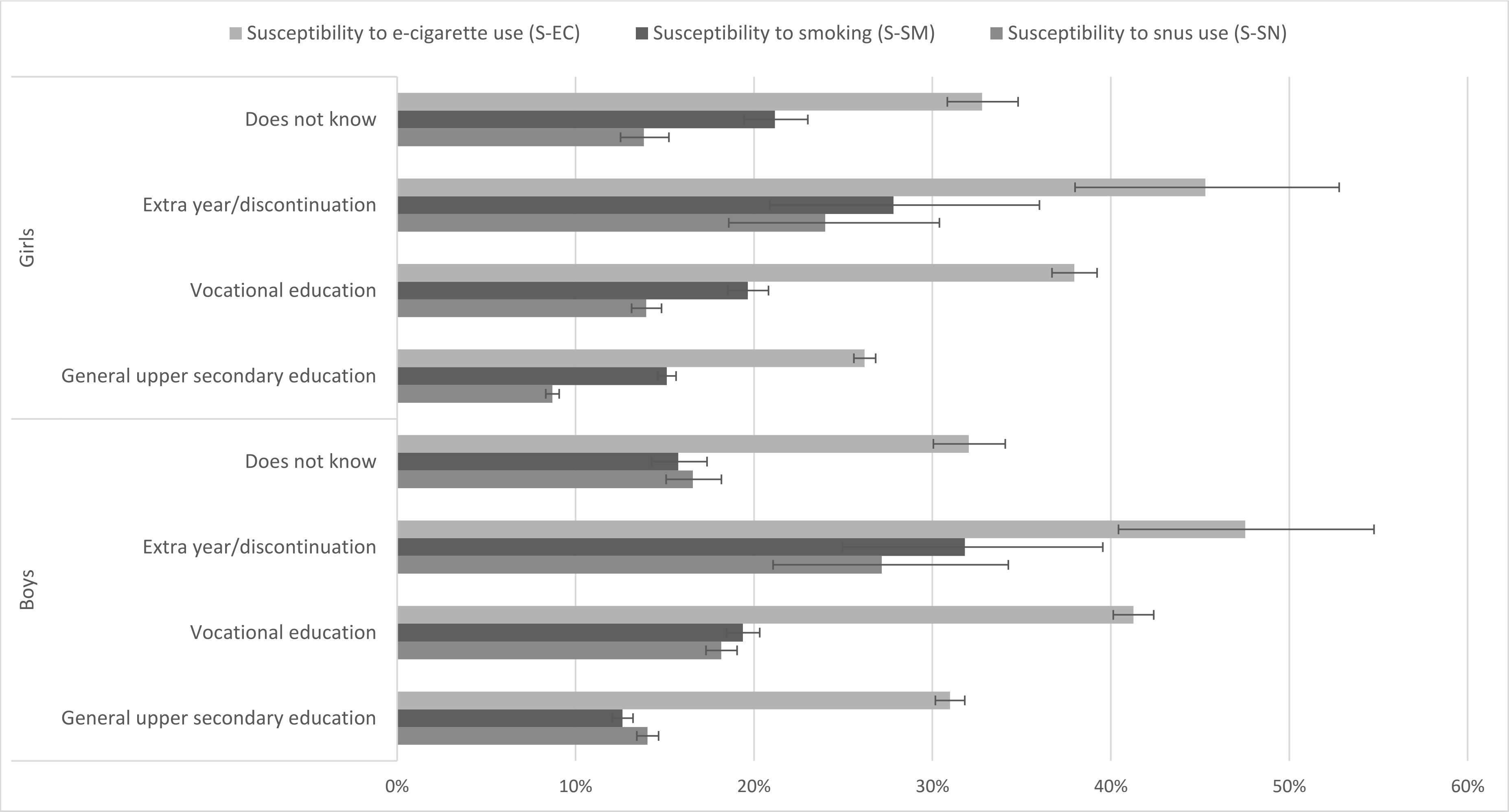

Supplement: ckae107_Supplementary_Data [file ckae107_supplementary_data.pdf]
